# Maximal diameter of liver abscess independently predicts prolonged hospitalization and poor prognosis in patients with pyogenic liver abscess

**DOI:** 10.1186/s12879-021-05873-7

**Published:** 2021-02-11

**Authors:** Chang Hun Lee, Hoon Gil Jo, Eun Young Cho, Jae Sun Song, Gum Mo Jung, Yong Keun Cho, Seung Young Seo, Seong Hun Kim, Sang Wook Kim, Seung Ok Lee, Soo Teik Lee, In Hee Kim

**Affiliations:** 1 Division of Gastroenterology, Department of Internal Medicine, Jeonbuk National University Medical School and Research Institute of Clinical Medicine of Jeonbuk National University Hospital-Jeonbuk National University Medical School, 20 Geonjiro, Dukjingu, Jeonju, Jeonbuk 54907 South Korea; 2 Division of Gastroenterology, Department of Internal Medicine, Wonkwang University College of Medicine and Hospital, Iksan, South Korea; 3 Division of Gastroenterology, Department of Internal Medicine, Presbyterian Medical Center, Jeonju, South Korea

**Keywords:** Liver abscess, pyogenic, Hospitalization, Mortality, in-hospital

## Abstract

**Background/aims:**

This study aimed to investigate the factors associated with prolonged hospital stay and in-hospital mortality in patients with pyogenic liver abscess.

**Methods:**

We retrospectively reviewed data from patients with pyogenic liver abscess who were admitted between 2005 and 2018 at three tertiary hospitals in Jeonbuk province, South Korea. Prolonged hospital stay was defined as a duration of hospital admission of more than 21 days.

**Results:**

A total of 648 patients (406 men and 242 women) diagnosed with pyogenic liver abscess were enrolled in the study. The mean maximal diameter of the liver abscess was 5.4 ± 2.6 cm, and 74.9% of the lesions were single. The three groups were divided according to the maximal diameter of the abscess. Laboratory parameters indicated a more severe inflammatory state and higher incidence of complications and extrahepatic manifestations with increasing abscess size. Rates of percutaneous catheter drainage (PCD) insertion, multiple PCD drainage, and salvage procedures as well as duration of drainage were also higher in the large liver abscess group. Of note, the duration of hospitalization and in-hospital mortality were significantly higher in the large hepatic abscess group. A multivariate analysis revealed that underlying diabetes mellitus, hypoalbuminemia, high baseline high-sensitivity C-reactive protein (hs-CRP) and procalcitonin levels, and large maximal abscess diameter were independent factors associated with prolonged hospital stay. Regarding in-hospital mortality, acute kidney injury at admission and maximal diameter of the abscess were independent factors associated with in-hospital mortality.

**Conclusions:**

A large maximal diameter of the liver abscess at admission indicated prolonged hospitalization and poor prognosis. More aggressive treatment strategies with careful monitoring are warranted in patients with large liver abscesses.

**Supplementary Information:**

The online version contains supplementary material available at 10.1186/s12879-021-05873-7.

## Background

Liver abscess is a disease in which the pathogen invades the liver, proliferates, and forms pus through the body’s inflammatory responses. It is the most common abscess in the abdominal cavity, and accounts for about half of all intraabdominal abscesses [1]. The incidence of liver abscess ranges from 2.30–17.59 per 100,000 individuals every year and is increasing worldwide [1–4]. Age, antibiotic use, comorbid diseases, such as diabetes and underlying hepatobiliary disease, and regular use of proton-pump inhibitors may facilitate the incidence of liver abscess [5–7]. It has become an emerging infectious disease, especially in Southeast Asia [8, 9].

The epidemiology of liver abscess has changed over the past several decades due to several factors such as economic status, hygiene practices, the increasing elderly population and associated underlying comorbid diseases, and increased incidence of hepatobiliary procedures [10, 11]. *Escherichia coli* is the most common causative organism; however, infections due to *Klebsiella pneumoniae* have been rising in recent decades. Direct contact via the biliary tract, leakage from the intestine, and hematogenous spreading can be routes for infection associated with liver abscess. A meta-analysis also showed an association between colorectal cancer and liver abscess [12].

Antibiotic treatment and proper drainage are the mainstay treatments for liver abscess. The American Society of Infectious Diseases recommends intravenous antibiotics as first-line therapies for complicated intraabdominal infections [13]. The duration of therapy with intravenous antibiotics for patients with pyogenic liver abscess is generally 2–3 weeks, followed by 1–2 months of oral therapy [14, 15]. The size of the liver abscess is usually used to determine whether image-guided needle aspiration, percutaneous catheter drainage, or surgical drainage should be performed. Some previous studies showed that catheter drainage was more effective than aspiration therapy in cases of liver abscess size ≥5 cm [16, 17]. In cases of abscesses of size > 10 cm, usually termed giant abscesses, it has been advised that drainage should be performed with caution because of the high incidence of complications [18, 19]. As proper antibiotic treatment and percutaneous drainage have become the main treatment options, mortality rates have been decreasing; however, recent mortality rates have been found to be 2–12%, depending on the medical environment [2, 3, 20, 21].

Although there have been several reports on hepatic abscess treatment and clinical outcomes, there are insufficient real-world data regarding clinical courses and outcomes related to the liver abscess size. To address these gaps in the literature, we evaluated the clinical characteristics of patients with liver abscess according to the maximal diameter of the abscess and analyzed the factors associated with prolonged hospitalization and prognosis.

## Methods

### Study subjects

In this cohort study, we reviewed the medical records of patients with liver abscess from January 2005 to December 2018 at three tertiary hospitals (Jeonbuk National University Hospital, Wonkwang University Hospital, and Presbyterian Medical Center) in Jeonbuk province, South Korea. Eligible patients were selected using the KCD code (K75, hepatic abscess, including subgroups) and checking computed tomography (CT) images to determine whether the diagnosis was compatible with pyogenic liver abscess. We included only patients with community-acquired pyogenic liver abscess and excluded patients with hospital-acquired infection [22]. A total of 648 patients were enrolled in this study. Demographic, laboratory, and clinical parameters were assessed and analyzed. Laboratory values were obtained twice; on admission and 1 week after. CT characteristics, complications, and features of the invasive procedure were also analyzed.

This study protocol was consistent with the ethical guidelines of the 1975 Declaration of Helsinki and was approved by the institutional review board of the Jeonbuk National University Hospital (approval number: CUH 2019–05-009). Written informed consent was waived by the institutional review board of the Jeonbuk National University Hospital due to the retrospective nature of the study.

### Data collection and definitions

Prolonged hospitalization was defined as a duration of hospital admission of more than 21 days, taking into consideration findings from previous research and mean hospital stays. Microbiologic etiologies were divided into monomicrobial or polymicrobial in accordance with single or multiple isolated organisms, respectively, from the abscess and/or blood culture. Invasive syndrome was defined as *Klebsiella pneumoniae* isolated from a liver abscess with metastatic infection, including septic embolism, meningitis, and endophthalmitis [23]. Regarding CT characteristics, multiloculation of the abscess was defined as presence of ≥1-mm-thick septations within the abscess pocket. Cystic appearance was defined as more than 50% of the hypodense or liquefied abscess cavity with an attenuation value less than 20 Hounsfield units [24]. Acute kidney injury (AKI) was defined based on the Kidney Disease Improving Global Outcomes Clinical Practice Guidelines criteria [25].

### Treatments

All patients were administered intravenous antibiotic therapy for the treatment of their abscesses. Invasive procedures, such as needle aspiration, catheter drainage, or surgical management, were also included as treatment options. In general, an abscess ≥ 3 cm with a predominantly cystic appearance was considered an indication for drainage. Image-guided needle aspiration was performed using 18-gauge disposable trocar needles of variable length. After abscess pocket puncture and serial dilatations, multipurpose drainage catheter of 8.5 Fr, 10.2 Fr, or 12.0 Fr (Cook, Bloomington, Ind, USA) was inserted to the abscess pocket based on the assessment by interventional specialists for drainage. PCD was maintained until minimal drainage amount, and no remnant abscess was observed. Salvage procedures include additional needle aspiration, PCD reposition, or salvage drainage of the abscess pocket. Patients were discharged if they showed clinical and biochemical improvement and could receive oral antibiotics after removal of PCD.

### Statistical analysis

Data are presented as means ± standard deviations or numbers (percentages). We compared continuous or categorical variables between the groups using the t-test, chi-square test, and ANOVA test. Factors associated with prolonged hospitalization and in-hospital mortality were analyzed using binary logistic regression analysis. Cumulative rates of in-hospital mortality were evaluated by the Kaplan-Meier method and compared using the log-rank test. The results were analyzed using the statistical software package IBM SPSS Statistics software (version 23.0, IBM Corporation, Armonk, NY). All significance tests were two-tailed, and *P*-values < 0.05 were considered statistically significant.

## Results

### Demographic and baseline clinical characteristics of the study population

The baseline characteristics of the 648 patients with liver abscess according to the maximal abscess size are summarized in Table 1. The mean age of the patients was 65.7 years, 406 (62.7%) were male, and there were no age or sex differences according to the size of the abscess. Additionally, the distribution of patients with underlying diseases did not differ according to the size of the liver abscess. Laboratory results showed that inflammatory markers, such as white blood cells, erythrocyte sedimentation rate, and high-sensitivity C-reactive protein (hs-CRP) levels were higher in patients with large liver abscesses; conversely, procalcitonin (PCT) levels were lower. The large liver abscess group had lower hemoglobin and albumin levels and higher aspartate aminotransferase and alanine aminotransferase (ALT) levels. From CT findings, approximately three-quarters of liver abscesses were single (74.4%), and more than half of them manifested cystic and multiloculated appearance. Furthermore, the proportion of single, cystic, and multiloculated abscesses were higher in the large abscess group.

**Table 1:** Demographic and baseline clinical characteristics of patients

Characteristics	Total (*n* = 648)	Maximal size of abscess			*P* value
		< 5 cm (*n* = 293)	5 ~ 10 cm (*n* = 312)	≥ 10 cm (*n* = 43)	
Age, years	65.7 ± 14.6	65.5 ± 14.9	66.4 ± 14.3	62.2 ± 14.7	0.658
Male sex	406 (62.7)	188 (64.2)	190 (60.9)	28 (65.1)	0.668
BMI, kg/m^2^	23.8 ± 3.5	23.7 ± 3.4	24.0 ± 3.5	23.1 ± 3.7	0.850
Significant alcohol drinking	58 (9.0)	29 (9.9)	25 (8.0)	4 (9.3)	0.717
Underlying disease					
Malignancy	95 (14.7)	48 (16.4)	41 (13.1)	6 (14.0)	0.525
Biliary disease	170 (26.2)	81 (27.6)	81 (26.0)	8 (18.6)	0.448
Diabetes mellitus	183 (28.2)	85 (29.0)	82 (26.3)	16 (37.2)	0.304
Hypertension	212 (32.7)	101 (34.5)	102 (32.7)	9 (20.9)	0.210
Chronic liver disease	32 (4.9)	15 (5.1)	14 (4.5)	3 (7.0)	0.765
Previous liver abscess	10 (1.5)	5 (1.7)	5 (1.6)	0 (0.0)	0.693
Vital sign at admission					
Decreased mentality	16 (2.6)	8 (2.9)	8 (2.7)	0 (0.0)	0.560
Systolic BP, mmHg	117.4 ± 22.5	118.6 ± 23.1	116.2 ± 21.8	117.5 ± 23.0	0.314
Diastolic BP, mmHg	71.5 ± 13.0	72.2 ± 13.1	70.7 ± 13.1	72.4 ± 12.4	0.391
**Body temperature, °C**	**37.3 ± 1.0**	**37.4 ± 1.1**	**37.2 ± 0.9**	**37.2 ± 0.9**	**0.003**
Heart rates, /min	88.2 ± 16.2	88.3 ± 16.1	87.7 ± 16.2	91.5 ± 16.7	0.647
Respiratory rates, /min	19.3 ± 2.0	19.3 ± 2.0	19.2 ± 2.1	19.6 ± 2.0	0.799
Laboratory findings					
**WBC, /mm**^**3**^	**13.8 ± 6.9**	**13.0 ± 7.3**	**14.0 ± 6.3**	**16.7 ± 6.9**	**0.001**
**Hemoglobin, g/dL**	**12.0 ± 2.0**	**12.2 ± 2.0**	**11.9 ± 1.9**	**11.0 ± 2.0**	**0.001**
**Platelet, × 1000/mm**^**3**^	**273.3 ± 221.8**	**239.6 ± 140.4**	**303.4 ± 283.5**	**285.1 ± 134.4**	**0.009**
**ESR, mm/hr**	**65.9 ± 31.7**	**59.2 ± 31.2**	**70.4 ± 30.4**	**77.1 ± 35.6**	**< 0.001**
**PT, INR**	**1.2 ± 0.2**	**1.2 ± 0.2**	**1.2 ± 0.2**	**1.3 ± 0.1**	**0.001**
Na, mmol/L	135.7 ± 4.6	135.7 ± 4.6	135.8 ± 4.6	134.6 ± 4.7	0.542
**AST, IU/L**	**94.8 ± 152.0**	**87.6 ± 147.6**	**93.0 ± 148.0**	**157.8 ± 194.6**	**0.040**
**ALT, IU/L**	**83.8 ± 105.3**	**80.0 ± 109.5**	**80.3 ± 88.2**	**134.2 ± 163.8**	**0.045**
**Total bilirubin, mg/dL**	**1.4 ± 1.6**	**1.6 ± 1.9**	**1.3 ± 1.3**	**1.2 ± 1.0**	**0.029**
**Albumin, g/dL**	**3.4 ± 0.5**	**3.5 ± 0.5**	**3.3 ± 0.5**	**3.0 ± 0.4**	**< 0.001**
Creatinine, mg/dL	1.0 ± 0.9	1.0 ± 0.8	1.0 ± 0.9	1.0 ± 1.2	0.637
LD, IU/L	582.0 ± 297.9	569.8 ± 303.3	576.7 ± 298.3	694.4 ± 240.5	0.110
**hs-CRP, mg/L**	**160.3 ± 84.6**	**139.8 ± 85.3**	**174.9 ± 81.0**	**193.3 ± 75.2**	**< 0.001**
**PCT, ng/mL**	**17.4 ± 28.6**	**19.9 ± 30.2**	**17.3 ± 28.8**	**4.6 ± 9.3**	**0.042**
CT findings					
**Non-single lesion**	**166 (25.6)**	**88 (30.0)**	**73 (23.4)**	**5 (11.6)**	**0.016**
**Cystic appearance**	**334 (51.5)**	**99 (33.8)**	**198 (63.5)**	**37 (86.0)**	**< 0.001**
**Multiloculated abscess**	**391 (60.3)**	**119 (40.6)**	**239 (76.6)**	**33 (76.7)**	**< 0.001**

Data were expressed as number (percentage) or mean ± standard deviation. *BMI* Body mass index, *BP* Blood pressure, *WBC* White blood cell, *ESR* Erythrocyte sedimentation rate, *PT* Prothrombin time, *Na* Sodium, *AST* Aspartate aminotransferase, *ALT* Alanine aminotransferase, *LD* Lactate dehydrogenase, *hs-CRP* High sensitivity C-reactive protein, *PCT* Procalcitonin, *CT* Computed tomography

### Characteristics of pyogenic liver abscess and clinical outcomes

The characteristics of the pyogenic liver abscesses are summarized in Table 2. In total, 12.3% of the patients had liver abscess-related intrahepatic complications, and 42.6% had extrahepatic manifestations. Regarding pleural effusion, more than half of the patients (50.6%) had pleural effusion in both pleural spaces, and effusion on the right side was more frequent than on the left side (42.4% vs. 7.0%, respectively). Invasive syndrome only accounted for 1.2% of the cases; these included eight cases of pulmonary septic embolism and five cases of endophthalmitis. Interestingly, the incidence of complications and extrahepatic manifestations was higher in patients with large liver abscesses. As a complication, the number of cases of abscess rupture was significantly higher in the large abscess group. In addition, the large abscess group had significantly higher incidence estimates of pleural effusion, ascites, and invasive syndrome.

**Table 2:** Progression and clinical outcomes of pyogenic liver abscess

Characteristics	Total (*n* = 648)	Maximal size of abscess			*P* value
		< 5 cm (*n* = 293)	5 ~ 10 cm (*n* = 312)	≥ 10 cm (*n* = 43)	
Complications					
**Rupture**	**18 (2.8)**	**6 (2.0)**	**8 (2.6)**	**4 (9.3)**	**0.025**
Hematoma	10 (1.5)	2 (0.7)	6 (1.9)	2 (4.7)	0.108
Biloma	13 (2.0)	5 (1.7)	5 (1.6)	3 (7.0)	0.055
Venous thrombosis	22 (7.5)	21 (6.7)	4 (9.3)	47 (7.3)	0.809
Extrahepatic manifestations					
Pulmonary edema	88 (13.6)	31 (10.6)	49 (15.7)	8 (18.6)	0.112
**Pleural effusion**	**207 (31.9)**	**73 (24.9)**	**110 (35.3)**	**24 (55.8)**	**< 0.001**
**Ascites**	**116 (17.9)**	**40 (13.7)**	**60 (19.2)**	**16 (37.2)**	**0.001**
**Invasive syndrome**	**13 (2.0)**	**6 (2.0)**	**4 (1.3)**	**3 (7.0)**	**0.044**
**Antibiotic treatment only**	**270 (41.7)**	**148 (50.5)**	**111 (35.6)**	**11 (25.6)**	**< 0.001**
Invasive procedure					
**Needle aspiration alone**	**12 (1.9)**	**12 (4.1)**	**0 (0.0)**	**0 (0.0)**	**< 0.001**
**PCD insertion**	**366 (56.5)**	**133 (45.4)**	**201 (64.4)**	**32 (74.4)**	**< 0.001**
**PCD insertion within 3 days**	**269 (41.5)**	**91 (31.1)**	**149 (47.8)**	**29 (67.4)**	**0.037**
**Multiple PCD drainage**	**78 (12.0)**	**28 (9.6)**	**36 (11.5)**	**14 (32.5)**	**< 0.001**
**Salvage procedure**	**235 (36.3)**	**65 (22.2)**	**143 (45.8)**	**27 (62.8)**	**< 0.001**
**Duration of drainage, days**	**12.6 ± 8.4**	**10.7 ± 5.5**	**12.1 ± 7.3**	**20.6 ± 14.3**	**< 0.001**
**Admission days, days**	**19.0 ± 12.1**	**17.4 ± 11.6**	**19.7 ± 11.3**	**24.0 ± 18.0**	**< 0.001**
**In-hospital expire**	**14 (2.2)**	**4 (1.4)**	**5 (1.6)**	**5 (11.6)**	**< 0.001**

Data were expressed as number (percentage) or mean ± standard deviation. *PCD* Percutaneous catheter drainage; Salvage procedure includes aspiration, catheter adjustment, and salvage drainage

About half of the patients were administered conservative therapy using intravenous antibiotics alone. Ultrasound-guided needle aspiration or PCD insertion was performed and there were no cases of surgical management. In the case of needle aspiration, only 4% of patients with small liver abscess (≤ 5 cm) underwent the procedure. PCD was inserted in about half of the patients, and the procedures were carried out within 3 days of admission in three-quarters of the patients. The rates of PCD insertion and the proportions of patients who underwent early PCD within 3 days of admission were higher in the large liver abscess group. In particular, the rates of multiple PCD drainage and salvage procedures as well as duration of drainage were also significantly higher in the large liver abscess. Of note, the mean admission period was 19.0 days, in-hospital mortality rate was 2.2% (14 patients), duration of hospitalization was longer, and prognosis was significantly worse in patients with large abscesses.

When we divided the patients into two groups based on the duration of admission with a cut-off value of 21 days, patients who were hospitalized for more than 21 days showed higher rates of underlying malignancy, diabetes mellitus (DM), and hypertension (Table S1). In addition, the prevalence of complications and extrahepatic manifestations was higher in patients with prolonged hospitalization. The rate of percutaneous catheter drainage (PCD) insertion, multiple PCD drainage, and salvage procedures as well as duration of drainage were also higher in the prolonged hospitalization group. Importantly, the mean maximal diameter of the liver abscess was larger in the prolonged hospitalization group.

We further evaluated the laboratory parameters 1 week after treatment according to the maximal diameter of the abscess. We noted that the level of inflammatory markers was still high, while that of albumin was statistically and significantly lower in patients with large liver abscesses (Table S2).

### Profiles of isolated microorganisms

Among the patients, 326 (50.4%) patients (total 365 isolations) showed positive culture results, and polymicrobial strains were isolated in 36 (5.6%) patients (Table 3). The *Klebsiella pneumoniae* species was the most common etiologic microorganism isolated 36.4% of all patients. Its distribution seemed to be low, and that of gram-positive bacteria seemed to be high in the large abscess group, though not significantly different. Additional antibiotic sensitivity results for the isolated microorganisms are shown in Table S3. There were 27 extended-spectrum β-lactamase (ESBL) positive cases―9 *Klebsiella pneumoniae*, 12 *Escherichia coli*, 2 *Aeromonas*, 2 *Enterobacter*, and 2 *Pseudomonas* species. Only 3.8% of *Klebsiella pneumoniae* strains exhibited ESBL positivity, while, other strains showed higher rates of ESBL positivity.

**Table 3:** Profiles of isolated microorganisms in patients with pyogenic liver abscess

Characteristics	Total (*n* = 648)	Maximal size of abscess			*P* value
		< 5 cm (*n* = 293)	5 ~ 10 cm (*n* = 312)	≥ 10 cm (*n* = 43)	
Positive culture results	326 (50.4)	147 (50.2%)	158 (50.8%)	21 (48.8%)	0.966
Monomicrobial	290 (44.8)	131 (44.7)	139 (44.6)	20 (46.5)	0.971
Polymicrobial	36 (5.6)	16 (5.5)	19 (6.1)	1 (2.3)	0.598
Isolated microorganisms					
Gram (−) organisms					
*Klebsiella pneumoniae*	236 (36.4)	106 (36.2)	118 (37.8)	12 (27.9)	0.445
*Escherichia coli*	34 (5.2)	16 (5.5)	16 (5.1)	2 (4.7)	0.967
*Aeromonas* species	2 (0.3)	1 (0.3)	1 (0.3)	0 (0.0)	0.930
*Enterobacter* species	15 (2.3)	8 (2.7)	6 (1.9)	1 (2.3)	0.804
*Acinetobacter* species	2 (0.3)	0 (0.0)	2 (0.6)	0 (0.0)	0.340
*Pseudomonas* species	9 (1.4)	4 (1.4)	5 (1.6)	0 (0.0)	0.701
*Citrobacter* species	2 (0.3)	0 (0.0)	2 (0.6)	0 (0.0)	0.340
Others	6 (0.9)	3 (1.0)	2 (0.6)	1 (2.3)	0.542
Gram (+) organisms					
*Streptococcus* species	30 (4.6)	13 (4.4)	14 (4.5)	3 (7.0)	0.750
*Staphylococcus* species	20 (3.1)	8 (2.7)	9 (2.9)	3 (7.0)	0.310
*Enterococcus* species	7 (1.1)	2 (0.7)	5 (1.6)	0 (0.0)	0.427
*Clostridium* species	1 (0.2)	1 (0.3)	0 (0.0)	0 (0.0)	0.545
Others	3 (0.5)	2 (0.7)	1 (0.3)	0 (0.0)	0.725
Fungus	1 (0.2)	0 (0.0)	1 (0.3)	0 (0.0)	–

Data were expressed as number (percentage) or mean ± standard deviation. *PCD* Percutaneous catheter drainage

### Findings of colonoscopic examination

Colonoscopic examination was performed in 91 patients around 3 months after hospital admission. Among the screened patients, 52.7% had some kind of lesion in the colon. The detection of colonic malignancy, colon polyp, or colonic diverticulum did not differ according to the size of the liver abscess (Table S4).

### Factors associated with prolonged hospitalization

We evaluated factors associated with prolonged hospitalization, i.e., hospitalization for more than 21 days. In the univariate analysis, the male sex, underlying malignancy, DM, hypertension, elevated serum ALT levels, decreased serum albumin levels, increased hs-CRP and PCT levels, maximal diameter of abscess, PCD insertion, and culture positivity were associated with prolonged hospitalization. The multivariate analysis revealed that underlying DM, hypoalbuminemia, high baseline hs-CRP and PCT levels, and large maximal diameter of abscess were independent factors associated with prolonged hospital stay (Table 4).

**Table 4:** Factors associated with prolonged hospital stay (≥ 21 days)

	Univariate analysis				Multivariate analysis			
	*P* value	OR	Lower CI	Upper CI	*P* value	OR	Lower CI	Upper CI
Age ≥ 65 years, vs < 65	0.208	1.24	0.89	1.72				
Male sex	**0.023**	**0.68**	**0.49**	**0.95**	0.762	0.912	0.501	1.659
Malignancy	**0.006**	**1.88**	**1.19**	**2.96**	0.139	1.784	0.829	3.835
Biliary disease	0.884	1.03	0.71	1.49				
**DM**	**0.005**	**1.67**	**1.17**	**2.38**	**0.019**	**2.018**	**1.120**	**3.634**
HTN	**0.032**	**1.45**	**1.03**	**2.05**	0.478	1.234	0.690	2.207
Significant alcohol consumption	0.667	0.88	0.49	1.55				
Decreased mentality at admission	0.993	1.00	0.31	2.95				
Shock at admission	0.285	1.51	0.70	3.19				
WBC ≥ 12,000 /mm^3^, at baseline	0.129	1.29	0.93	1.80				
Hb < 10 mg/dL, at baseline	0.196	1.35	0.85	2.11				
Na < 130 mmol/L, at baseline	0.534	1.16	0.72	1.85				
ALT ≥ 200 IU/L, at baseline	**0.012**	**2.23**	**1.20**	**4.19**	0.459	1.493	0.517	4.315
**Albumin < 3 g/dL**	**0.002**	**1.875**	**1.253**	**2.805**	**0.048**	**2.009**	**1.007**	**4.009**
**hs-CRP ≥ 200 mg/L, at baseline**	**< 0.001**	**1.91**	**1.35**	**2.70**	**0.024**	**2.008**	**1.094**	**3.684**
**PCT ≥ 5 ng/mL, at baseline**	**0.009**	**1.99**	**1.19**	**3.34**	**0.041**	**1.946**	**1.029**	**3.680**
Non-single abscess lesion	0.134	1.33	0.91	1.92				
**Maximal abscess diameter (< 5 cm, 5 ~ 10 cm, ≥10 cm)**	**< 0.001**	**1.84**	**1.40**	**2.44**	**0.007**	**1.962**	**1.206**	**3.191**
PCD insertion during admission	**< 0.001**	**1.82**	**1.30**	**2.56**	0.093	1.835	0.903	3.732
PCD insertion within 3 days of admission	0.114	0.68	0.43	1.10				
Culture positive (blood or pus)	**0.001**	**1.75**	**1.26**	**2.44**	0.310	1.355	0.754	2.437
*Klebsiella pneumoniae* isolation	0.068	1.364	0.978	1.902				
SIRS criteria at admission	0.065	1.16	0.99	1.36				
qSOFA score ≥ 2 at admission	0.756	1.22	0.31	4.33				
AKI at admission	**0.011**	**1.760**	**1.141**	**2.714**	0.363	0.708	0.337	1.490

*DM* Diabetes mellitus, *HTN* Hypertension, *WBC* White blood cells, *Hb* Hemoglobin, *Na* Sodium, *ALT* Alanine aminotransferase, *hs-CRP* High sensitivity C-reactive protein, *PCT* Procalcitonin, *PCD* Percutaneous catheter drainage, *SIRS* Systemic inflammatory response syndrome, *qSOFA* Quick sepsis-related organ failure assessment, *AKI* Acute kidney injury, *OR* Odd ratio, *CI* Confidence interval

### Factors associated with in-hospital mortality

**Fig. 1:**
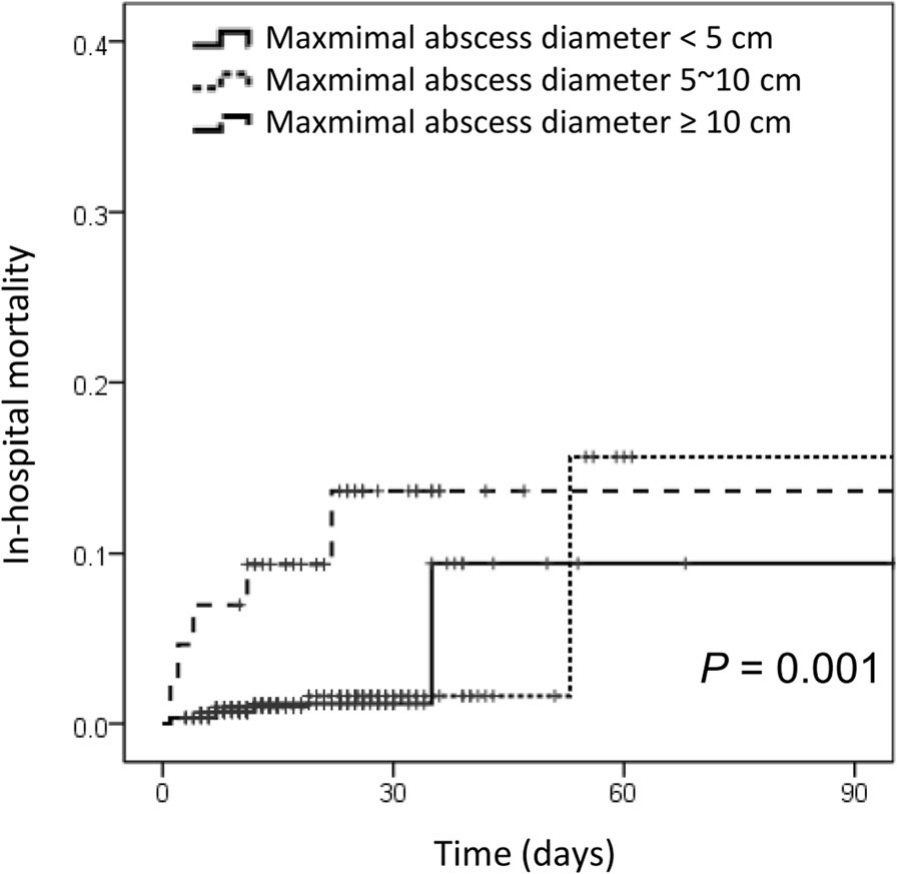
Comparison of survival rates using Kaplan-Meier analysis according to the maximal diameter of liver abscess

Regarding in-hospital mortality, 2.2% of the patients (14 patients) died during the study period. In the univariate analysis, old age, underlying malignancy, biliary disease, mental deterioration at admission, leukocytosis, anemia, high serum ALT level, hypoalbuminemia, elevated hs-CRP level, number of liver abscesses, maximal diameter of the liver abscess, quick sepsis-related organ failure assessment score > 2 at admission, and AKI at admission were related to mortality in this study. In the multivariate analysis, AKI at admission and maximal diameter of the abscess were independent factors associated with in-hospital mortality (Table 5). The Kaplan-Meier survival analysis revealed that the cumulative incidence of in-hospital mortality was affected by maximal diameter of the liver abscess, and this effect was statistically significant (Fig. 1).

**Table 5:** Factors associated with in-hospital mortality

	Univariate analysis				Multivariate analysis			
	*P* value	OR	Lower CI	Upper CI	*P* value	OR	Lower CI	Upper CI
Age ≥ 65 years, vs < 65	**0.046**	**1.63**	**1.25**	**29.88**	0.969	0.963	0.146	6.341
Male sex	0.889	1.07	0.37	3.53				
Malignancy	**0.006**	**4.59**	**1.48**	**13.52**	0.120	3.581	0.717	17.892
Biliary disease	**0.013**	**3.88**	**1.33**	**11.96**	0.133	0.294	0.060	1.450
DM	0.569	0.69	0.15	2.23				
HTN	0.369	0.55	0.12	1.80				
Decreased mentality at admission	**0.016**	**6.99**	**1.03**	**28.97**	0.285	0.126	0.003	5.621
Shock at admission	0.131	3.28	0.50	12.78				
WBC ≥ 12,000 /mm^3^, at baseline	**0.035**	**5.03**	**1.36**	**32.47**	0.743	0.747	0.131	4.271
Hb < 10 mg/dL, at baseline	**0.009**	**4.88**	**1.30**	**15.21**	0.221	0.370	0.075	1.822
Na < 130 mmol/L, at baseline	0.405	1.73	0.39	5.69				
ALT ≥200 IU/L, at baseline	**0.005**	**5.50**	**1.46**	**17.20**	0.095	4.582	0.766	27.406
Albumin < 3 g/dL	**0.002**	**5.700**	**1.942**	**16.734**	0.061	4.284	0.937	19.591
hs-CRP ≥ 200 mg/L, at baseline	**0.029**	**3.53**	**1.16**	**11.82**	0.055	4.776	0.969	23.543
PCT ≥ 10 ng/mL, at baseline	0.069	7.73	1.12	152.44				
Non-single abscess lesion	**0.044**	**2.99**	**1.01**	**8.85**	0.109	0.293	0.065	1.312
**Maximal abscess diameter (< 5 cm, 5 ~ 10 cm, ≥10 cm)**	**0.006**	**3.221**	**1.397**	**7.423**	**0.037**	**3.399**	**1.075**	**10.741**
PCD insertion during admission	0.304	0.57	0.19	1.66				
PCD insertion within 3 days of admission	0.587	1.82	0.29	35.07				
Culture positive (blood or pus)	0.111	0.386	0.120	1.245				
*Klebsiella pneumoniae* isolation	0.254	0.473	0.131	1.711				
SIRS criteria at admission	0.078	1.54	0.94	2.50				
qSOFA score ≥ 2 at admission	**0.005**	**10.40**	**1.50**	**45.28**	0.566	0.358	0.011	11.968
**AKI at admission**	**< 0.001**	**22.708**	**6.212**	**83.016**	**0.001**	**12.023**	**2.639**	**54.786**

*DM* Diabetes mellitus, *HTN* Hypertension, *WBC* White blood cells, *Hb* Hemoglobin, *Na* Sodium, *ALT* Alanine aminotransferase, *hs-CRP* High sensitivity C-reactive protein, *PCT* Procalcitonin, *PCD* Percutaneous catheter drainage, *SIRS* Systemic inflammatory response syndrome, *qSOFA* Quick sepsis-related organ failure assessment, *AKI* Acute kidney injury, *OR* Odd ratio, *CI* Confidence interval

## Discussion

Liver abscess is the most common type of abscess found in intraabdominal organs and is of great concern because it causes high rates of morbidity and mortality. In the present study, we reviewed the clinical findings of pyogenic liver abscess according to the maximal size of the abscess. When divided into three groups according to abscess size, there was no difference in age, sex, underlying conditions, and causative microorganisms across the groups. However, the incidence of complications and extrahepatic manifestations of hepatic abscess as well as the serum levels of inflammatory markers were higher in patients with large hepatic abscesses. Moreover, rates of PCD insertion, multiple PCD drainage, and salvage procedures as well as duration of drainage were higher in the large liver abscess group. Most importantly, the maximal diameter of the liver abscess was an independent factor that was significantly associated with prolonged hospitalization and in-hospital mortality.

In recent decades, the incidence of pyogenic liver abscesses has increased in patients due to an increase in risk factors worldwide, especially in developed countries. In the present study, *Klebsiella pneumoniae* accounted for the highest number of infections, followed by *Escherichia coli.* These findings were very similar to those previously reported in the early 2000s in Southeast Asia, including in South Korea. Regarding antibiotic resistance, most *Klebsiella pneumoniae* species were antibiotic-sensitive; however, isolated *E.coli* revealed high positive results for ESBL. The prevalence of invasive syndrome was relatively lower than that in previous studies; these studies reported prevalence estimates of metastatic infections to be approximately 5–10% [15, 20, 26]. Regarding the size of the liver abscess, demographic and isolated microorganisms did not differ according to the size of the liver abscess. Underlying malignancy, DM, biliary tract diseases, and liver cirrhosis are known risk factors for liver abscess [5, 7, 27–30], and the present study covered a large number of individuals with these risk factors; however, the distribution of patients with these underlying diseases did not differ according to the size of the abscess. The detection rate of colonic lesions by colonoscopic examination also showed no difference according to the size of the abscess. These findings suggest that the size of the abscess is not solely related to patients’ underlying conditions or comorbid diseases.

The factor that showed the most significant difference according to the size of the liver abscess was the incidence of complications and extrahepatic manifestations. The statistical analyses showed that as the size of the abscess increased, the number of patients with abscess rupture, pleural effusion, ascites, and invasive syndrome also significantly increased. In particular, more than half of the patients in the giant liver abscess group presented with pleural effusion. This could be attributed to the large area of liver abscess, which may have a greater effect on the surrounding area and the systemic condition. This study also found that there was more severe inflammation associated with a large liver abscess. Inflammatory conditions can be related to the size of the liver abscess because when bacteria enter the body, the immune response is activated and facilitated, and white blood cells are recruited to fight the pathogen. This causes swelling at the site of the infection and the death of nearby tissues, which results in the formation of a pus-filled abscess cavity. The size of the cavity may be influenced by the bacterial load, severity of inflammation, and immune status.

The present study showed that the maximal diameter of the abscess, underlying DM, hypoalbuminemia, and high hs-CRP and PCT levels were independent factors related to prolonged hospitalization. Elevated hs-CRP and PCT levels indicate severe inflammation, and underlying DM reflects an immunocompromised state. With respect to a large liver abscess, the size of the liver abscess might be influenced by high bacterial load, severe inflammation, and immunocompromised state of the host. Additionally, more time may be needed to drain the pus, and parenteral antibiotics by themselves may be insufficient for treatment because of inadequate penetration of the antibiotics or ineffective medium for bacterial elimination [19]. A previous study reported that the risk of complications was relatively higher in patients with giant abscesses, which can also result in prolonged hospital stay. The present study showed a high frequency of PCD insertion with multiple drainage in the large abscess group with many patients underwent salvage procedures thereafter. Although large liver abscesses had a higher percentage of single and cystic lesions, they often required additional procedures such as multiple and salvage drainages. These conditions may affect the duration of abscess drainage.

Regarding treatment outcomes, mortality from liver abscess has decreased remarkably due to advances in medical technology and the use of proper antibiotics in conjunction with image-guided percutaneous drainage as the main treatment strategy. The mortality rate in the early 1900s was 60–80%; however, recent studies have reported a mortality rate of 2–13% [31]. According to previous studies, comorbid diseases and risk factors such as underlying DM, malignancy, or liver cirrhosis have been associated with poor prognosis [7, 32]. In the present study, in-hospital mortality was as low as 2%, and upon reviewing the cause of death, most patients died of sepsis. There were two factors that were independently associated with in-hospital mortality: AKI at the time of visit and the maximal diameter of the liver abscess. One of the most significant factors was accompanying AKI at admission, which may be because AKI tends to be an initial step in organ failure due to infection or sepsis. In particular, the size of the liver abscess by itself was independently correlated with in-hospital mortality.

These findings may be attributed to a few reasons. First, laboratory results showed higher levels of inflammatory markers that were maintained even 1 week after hospitalization. Severe inflammation may progress to septic conditions and multi-organ dysfunction. Second, the relatively higher rate of complications and extrahepatic manifestations may be related to prognosis. The present study revealed that there was a higher rate of liver abscess rupture, pleural effusion, ascites, and invasive syndrome in patients with large liver abscesses. A large abscess may adversely affect the adjacent environment and worsen the systemic condition, leading to death. Lastly, proper management of liver abscess may be difficult because of the size of the abscess. Insufficient catheter drainage due to the large abscess pocket with multiple septations or inadequate treatment response to antibiotics should be considered in current treatment strategies.

A limitation of this study was that it was a retrospective cohort study. Different baseline characteristics among groups might affect the results. In addition, several clinical factors, such as discharge criteria and indications for invasive procedures, may vary depending on the clinicians or operators. Lastly, this was a regional study and hence, did not reflect the clinical characteristics of the entire population. Conducting large-scale prospective studies to evaluate prolonged hospitalization and prognosis are warranted.

## Conclusions

In summary, the present study evaluated the current epidemiology and clinical characteristics of pyogenic liver abscess according to the maximal size of the liver abscess. Our study revealed that the maximal diameter of liver abscess was significantly associated with prolonged hospitalization and higher in-hospital mortality rate, which may be due to the higher incidence of complications and extrahepatic manifestations as well as severe inflammatory states in patients with large liver abscesses. These findings warrant a more aggressive treatment strategy with careful monitoring in patients with large liver abscesses.

## Supplementary Information

**Additional file 1: Table S1.** Demographic and clinical characteristics of patients among survivors. **Table S2.** Laboratory parameters after 1 week of treatment. **Table S3.** Profiles of isolated microorganisms and antibiotic resistance in patients with pyogenic liver abscess. **Table S4.** Colonoscopic examination findings.

## Data Availability

The datasets used and/or analysed during the current study are available from the corresponding author on reasonable request.

## Abbreviations

AKI: Acute kidney injury
ALT: Alanine aminotransferase
CT: Computed tomography
DM: Diabetes mellitus
PCD: Percutaneous catheter drainage
ESBL: Extended-spectrum-β-lactamase
ESR: Erythrocyte sedimentation rate
hs-CRP: High-sensitivity C-reactive protein
KCD: Korean standard classification of diseases
PCT: Procalcitonin
