# Supplementary material for: Maximal diameter of liver abscess independently predicts prolonged hospitalization and poor prognosis in patients with pyogenic liver abscess
Source: BMC Infect Dis. 2021 Feb 11;21:171. doi: 10.1186/s12879-021-05873-7 (PMC7879520; doi:10.1186/s12879-021-05873-7)
Supplement: Supplementary file 1 — Additional file 1: Table S1. Demographic and clinical characteristics of patients among survivors. Table S2. Laboratory parameters after 1 week of treatment. Table S3. Profiles of isolated microorganisms and antibiotic resistance in patients with pyogenic liver abscess. Table S4. Colonoscopic examination findings. [file 12879_2021_5873_MOESM1_ESM.docx]

**Maximal diameter of liver abscess independently predicts prolonged hospitalization and poor prognosis in patients with pyogenic liver abscess**

Chang Hun Lee^1,*^, Hoon Gil Jo^2,*^, Eun Young Cho^2^, Jae Sun Song^3^, Gum Mo Jung^3^, Yong Keun Cho^3^, Seung Young Seo^1^, Seong Hun Kim^1^, Sang Wook Kim^1^, Seung Ok Lee^1^, Soo Teik Lee^1^, and In Hee Kim^1,¶^

^1^Division of Gastroenterology, Department of Internal Medicine, Jeonbuk National University Medical School and Research Institute of Clinical Medicine of Jeonbuk National University Hospital-Jeonbuk National University Medical School, Jeonju, South Korea

^2^Division of Gastroenterology, Department of Internal Medicine, Wonkwang University College of Medicine and Hospital, Iksan, South Korea

^3^Division of Gastroenterology, Department of Internal Medicine, Presbyterian Medical Center, Jeonju, South Korea

^*^These authors (Chang Hun Lee and Hun Gil Jo) are equally contributed to this work.

Address correspondence to:

**^¶^In Hee Kim, MD, PhD.** Department of Internal Medicine, Jeonbuk National University Medical School, Research Institute of Clinical Medicine of Jeonbuk National University-Biomedical Research Institute of Jeonbuk National University Hospital, 20 Geonjiro, Dukjingu, Jeonju, Jeonbuk 54907, South Korea. Phone: +82-63-250-1677; Fax: +82-63-254-1609; E-mail: [ihkimmd@jbnu.ac.kr](mailto:ihkimmd@jbnu.ac.kr); Orcid ID:0000-0003-3863-7907

**Contents**

1. Supplementary Tables

**1. Supplementary Tables**

**Table S1. Demographic and clinical characteristics of patients among survivors.**

| Characteristics | Total  (n = 634) | Hospitalization  < 21 days  (n = 410) | Hospitalization  ≥ 21 days  (n = 224) | P value |
| --- | --- | --- | --- | --- |
| Age | 65.5 ± 14.6 | 64.9 ± 15.2 | 66.6 ± 13.4 | 0.155 |
| **Male sex** | **397 (62.6)** | **270 (65.9)** | **127 (56.7)** | **0.028** |
| BMI, m/kg^2^ | 23.8 ± 3.4 | 23.8 ± 3.3 | 23.8 ± 3.7 | 0.882 |
| Significant alcohol drinking | 58 (9.1) | 39 (9.5) | 19 (8.5) | 0.775 |
| Underlying diseases |  |  |  |  |
| **Malignancy** | **89 (14.0)** | **46 (11.2)** | **43 (19.2)** | **0.008** |
| Biliary disease | 162 (25.6) | 104 (25.4) | 58 (25.9) | 0.960 |
| **Diabetes mellitus** | **180 (28.4)** | **101 (24.6)** | **79 (35.3)** | **0.006** |
| **Hypertension** | **209 (33.0)** | **123 (30.0)** | **86 (38.4)** | **0.039** |
| Chronic liver disease | 31 (4.9) | 18 (4.4) | 13 (5.8) | 0.551 |
| Previous liver abscess | 10 (1.6) | 6 (1.5) | 4 (1.8) | 1.000 |
| Vital sign at admission |  |  |  |  |
| Decreased mentality | 14 (2.3) | 9 (2.3) | 5 (2.3) | 1.000 |
| Systolic BP, mmHg | 117.5 ± 22.4 | 118.2 ± 22.2 | 116.2 ± 23.0 | 0.279 |
| Diastolic BP, mmHg | 71.5 ± 13.0 | 72.1 ± 12.7 | 70.4 ± 13.5 | 0.111 |
| Body temperature, ℃ | 37.3 ± 1.0 | 37.2 ± 1.0 | 37.4 ± 1.1 | 0.193 |
| Heart rates, /min | 88.0 ± 16.0 | 87.3 ± 15.1 | 89.4 ± 17.4 | 0.137 |
| **Respiratory rates, /min** | **19.3 ± 2.0** | **19.1 ± 1.8** | **19.6 ± 2.2** | **0.010** |
| Laboratory findings |  |  |  |  |
| WBC, /mm^3^ | 13.6 ± 6.7 | 13.2 ± 6.3 | 14.4 ± 7.5 | 0.059 |
| **Hemoglobin, g/dL** | **12.0 ± 1.9** | **12.1 ± 1.9** | **11.8 ± 2.0** | **0.020** |
| Platelet, x1000/mm^3^ | 275.7 ± 223.5 | 280.7 ± 253.8 | 266.5 ± 154.8 | 0.445 |
| ESR, mm/hr | 66.0 ± 31.5 | 64.9 ± 32.5 | 68.0 ± 29.6 | 0.249 |
| PT, INR | 1.2 ± 0.2 | 1.2 ± 0.2 | 1.2 ± 0.2 | 0.064 |
| Na, mmol/L | 135.7 ± 4.6 | 135.8 ± 4.6 | 135.6 ± 4.7 | 0.575 |
| **AST, IU/L** | **91.4 ± 147.1** | **81.3 ± 149.7** | **110.0 ± 140.5** | **0.019** |
| ALT, IU/L | 81.7 ± 99.7 | 76.4 ± 103.7 | 91.4 ± 91.4 | 0.060 |
| **Total bilirubin, mg/dL** | **1.4 ± 1.6** | **1.3 ± 1.2** | **1.6 ± 2.1** | **0.031** |
| **Albumin, g/dL** | **3.4 ± 0.5** | **3.5 ± 0.5** | **3.3 ± 0.5** | **< 0.001** |
| Creatinine, mg/dL | 1.0 ± 0.7 | 1.0 ± 0.7 | 1.0 ± 0.7 | 0.175 |
| LD, IU/L | 575.0 ± 292.9 | 559.6 ± 303.9 | 601.2 ± 272.0 | 0.137 |
| **hs-CRP, mg/L** | **159.4 ± 84.5** | **150.1 ± 82.0** | **176.3 ± 86.4** | **< 0.001** |
| PCT, ng/mL | 17.4 ± 28.9 | 16.4 ± 27.0 | 19.4 ± 32.1 | 0.417 |
| Characteristics of abscess |  |  |  |  |
| Non-single lesion | 159 (25.1) | 95 (23.2) | 64 (28.6) | 0.160 |
| **Maximal diameter, cm** | **5.4 ± 2.6** | **5.0 ± 2.3** | **6.2 ± 2.8** | **< 0.001** |
| **Cystic appearance** | **328 (51.7)** | **194 (47.3)** | **134 (59.8)** | **0.003** |
| **Multiloculated abscess** | **385 (60.7)** | **234 (57.1)** | **151 (67.4)** | **0.014** |
| Complications |  |  |  |  |
| **Rupture** | **17 (2.7)** | **6 (1.5)** | **11 (4.9)** | **0.021** |
| **Hematoma** | **9 (1.4)** | **2 (0.5)** | **7 (3.1)** | **0.020** |
| **Biloma** | **13 (2.1)** | **3 (0.7)** | **10 (4.5)** | **0.004** |
| Venous thrombosis | 47 (7.4) | 27 (6.6) | 20 (8.9) | 0.359 |
| Extrahepatic manifestations |  |  |  |  |
| **Pulmonary edema** | **87 (13.7)** | **34 (8.3)** | **53 (23.7)** | **< 0.001** |
| **Pleural effusion** | **202 (31.9)** | **100 (24.4)** | **102 (45.5)** | **< 0.001** |
| **Ascites** | **110 (17.4)** | **48 (11.7)** | **62 (27.7)** | **< 0.001** |
| Invasive syndrome | 13 (2.1) | 5 (1.2) | 8 (3.6) | 0.088 |
| **Antibiotic treatment only** | **262 (41.3)** | **189 (46.1)** | **73 (32.6)** | **< 0.001** |
| Invasive procedure |  |  |  |  |
| **Needle aspiration alone** | 12 (1.9) | 9 (2.2) | 3 (1.3) | 0.652 |
| **PCD insertion** | **360 (56.8)** | **212 (51.7)** | **148 (66.1)** | **0.001** |
| PCD insertion within first 3 days | 264 (73.3) | 162 (76.4) | 102 (68.9) | 0.114 |
| **Multiple PCD drainage** | **76 (12.0)** | **30 (7.3)** | **46 (20.5)** | **< 0.001** |
| **Salvage procedure** | **230 (36.3)** | **110 (26.8)** | **120 (53.6)** | **< 0.001** |
| **Duration of drainage, days** | **12.7 ± 8.4** | **9.3 ± 7.2** | **17.1 ± 7.9** | **< 0.001** |
| Isolated microorganisms |  |  |  |  |
| **Positive culture results** | **322 (50.9)** | **188 (46.0)** | **134 (59.8)** | **0.001** |
| *Klebsiella pneumoniae* isolation | 232 (72.0) | 140 (74.5) | 92 (68.7) | 0.308 |

Data were expressed as number (percentage) or mean ± standard deviation. BMI, body mass index; BP, blood pressure; WBC, white blood cell; ESR, erythrocyte sedimentation rate; PT, prothrombin time; Na, sodium; AST, aspartate aminotransferase; ALT, alanine aminotransferase; LD, lactate dehydrogenase; hs-CRP, high sensitivity C-reactive protein; PCT, procalcitonin; PCD, percutaneous catheter drainage.

**Table S2. Laboratory parameters after 1 week of treatment.**

| Characteristics | Total  (n=648) | Maximal size of abscess | | | P value |
| --- | --- | --- | --- | --- | --- |
|  |  | < 5 cm  (n=293) | 5~10 cm  (n=312) | ≥ 10 cm  (n=43) |  |
| **WBC, /mm^3^** | **11.0 ± 4.9** | **9.8 ± 4.3** | **11.8 ± 5.3** | **13.2 ± 4.4** | **< 0.001** |
| **Hemoglobin, g/dL** | **11.4 ± 1.7** | **11.7 ± 1.6** | **11.3 ± 1.7** | **10.6 ± 1.6** | **< 0.001** |
| **Platelet, x1000/mm^3^** | **365.6 ± 156.0** | **350.5 ± 145.2** | **377.6 ± 165.5** | **380.8 ± 148.3** | **0.040** |
| ESR, mm/hr | 67.7 ± 27.3 | 64.9 ± 28.1 | 69.4 ± 25.9 | 72.0 ± 30.8 | 0.065 |
| **PT, INR** | **1.2 ± 0.2** | **1.2 ± 0.2** | **1.2 ± 0.2** | **1.3 ± 0.2** | **< 0.001** |
| Na, mmol/L | 137.1 ± 4.1 | 137.3 ± 3.8 | 137.0 ± 4.4 | 136.7 ± 3.9 | 0.205 |
| AST, IU/L | 38.4 ± 40.1 | 40.2 ± 47.6 | 36.2 ± 29.7 | 43.1 ± 49.9 | 0.618 |
| ALT, IU/L | 40.0 ± 36.2 | 43.9 ± 38.9 | 35.6 ± 29.0 | 46.8 ± 57.2 | 0.138 |
| Total bilirubin, mg/dL | 1.0 ± 1.9 | 1.1 ± 2.7 | 0.9 ± 1.1 | 0.8 ± 0.6 | 0.140 |
| **Albumin, g/dL** | **3.2 ± 0.5** | **3.3 ± 0.5** | **3.1 ± 0.5** | **2.9 ± 0.4** | **< 0.001** |
| Creatinine, mg/dL | 0.8 ± 0.7 | 0.8 ± 0.7 | 0.8 ± 0.7 | 0.6 ± 0.4 | 0.263 |
| **LD, IU/L** | **520.5 ± 210.3** | **480.1 ± 172.4** | **533.5 ± 201.1** | **668.4 ± 356.6** | **< 0.001** |
| **hs-CRP, mg/L** | **71.4 ± 60.2** | **57.0 ± 54.8** | **81.2 ± 62.6** | **94.9 ± 56.0** | **< 0.001** |

Data were expressed as mean ± standard deviation. WBC, white blood cell; ESR, erythrocyte sedimentation rate; PT, prothrombin time; Na, sodium; AST, aspartate aminotransferase; ALT, alanine aminotransferase; LD, lactate dehydrogenase; hs-CRP, high sensitivity C-reactive protein.

**Table S3.** **Profiles of isolated microorganisms and antibiotic resistance in patients with pyogenic liver abscess**

|  | Total  (n=365) | 3rd cephalo-  sporin | Ciprofloxacin or levofloxacin | Ampicillin or oxacillin | ESBL^*^ | Vanco-  mycin |
| --- | --- | --- | --- | --- | --- | --- |
| Gram (-) organisms |  |  |  |  |  | - |
| *Klebsiella pneumoniae* | 236 | 9/235 (3.8) | 12/227 (5.3) | 189/189 (100) | 9/233 (3.8) | - |
| *Escherichia coli* | 34 | 14/34 (41.2) | 17/32 (53.1) | 20/25 (80) | 12/33 (36.4) | - |
| *Aeromonas* species | 2 | 2 (100) | 2 (100) | 2 (100) | 2 (100) | - |
| *Enterobacter* species | 15 | 4 (26.7) | 4 (26.7) | 12 (80) | 2 (13.3) | - |
| *Acinetobacter* species | 2 | - | - | - | - |  |
| *Pseudomonas* species | 9 | 6 (66.7) | 6 (66.7) | 7 (77.8) | 5 (55.6) | - |
| *Citrobacter* species | 2 | 1 (50.0) | 0 (0.0) | 2 (100) | - | - |
| *Others* | 6 | - | - | - | - | - |
| Gram (+) organisms |  |  |  |  |  |  |
| *Streptococcus* species | 30 | 0/15 (0.0) | 0/14 (0.0) | 9/24 (37.5) | - | 0 (0.0) |
| *Staphylococcus* species | 20 | 2/13 (15.4) | 6/18 (33.3) | 8/17 (47.1) | - | 0 (0.0) |
| *Enterococcus* species | 7 | 0 (0.0) | 3/5 (60) | 5/6 (83.3) | - | 0 (0.0) |
| *Clostridium* species | 1 | - | - | - | - | - |
| *Others* | 3 | - | - | - | - | - |

Data were expressed as number (percentage).

^*^Extended spectrum β-lactamase

**Table S4. Colonoscopic examination findings**

| Characteristics | Total  (n = 91) | Maximal size of abscess | | | P value |
| --- | --- | --- | --- | --- | --- |
|  |  | < 5 cm  (n = 38) | 5~10 cm  (n = 46) | ≥ 10 cm  (n = 7) |  |
| Within normal range | 43 (47.3) | 20 (52.6) | 22 (47.8) | 1 (14.3) | 0.174 |
| Newly detected lesion |  |  |  |  |  |
| Colon malignancy | 5 (5.5) | 2 (5.3) | 2 (4.3) | 1 (14.3) | 0.559 |
| Colon polyp | 37 (40.7) | 11 (28.9) | 21 (45.7) | 5 (71.4) | 0.068 |
| Colonic diverticulum | 10 (11.0) | 3 (7.9) | 7 (15.2) | 0 (0.0) | 0.354 |

Data were expressed as number (percentage).
